# Long non-coding RNA SNHG7 serves as a diagnostic biomarker for acute coronary syndrome and its predictive value for the clinical outcome after percutaneous coronary intervention

**DOI:** 10.1186/s13019-024-02855-z

**Published:** 2024-07-16

**Authors:** Ran Liao, Qing Han, Li Zhang

**Affiliations:** Department of Cardiovascular Medicine, Jiujiang City Key Laboratory of Cell Therapy, JiuJiang NO.1 People’s Hospital, No. 48, Taling South Road, Xunyang District, Jiujiang, 332000 Jiangxi Province People’s Republic of China

**Keywords:** SNHG7, ACS, PCI, Diagnosis, Prognosis

## Abstract

**Background:**

Acute coronary syndrome (ACS) is one of the common causes of cardiovascular death. The related lncRNAs were novel approaches for early diagnosis and intervention. This paper focused on the clinical function of SNHG7 for patients after PCI.

**Methods:**

The expression of SNHG7 was assessed in ACS patients. The predictive roles of SNHG7 were unveiled by the ROC curve. The relationship between SNHG7 and Gensini scores was judged by Pearson analysis. One-year follow-up was conducted and all patients were catalogued into different groups based on the prognosis. The qRT-PCR, K-M curve, and Cox regression analysis were performed to document the prognostic significance of SNHG7.

**Results:**

SNHG7 was highly expressed in ACS and its three subtypes. SNHG7 showed a certain value in predicting ACS, UA, NSTEMI, and STEMI. Gensini is a closely correlated indicator of SNHG7. The declined expression of SNHG7 was observed in the non-MACE and survival groups. The risk of MACE and death was increased in the group with high expression of SNHG7. SNHG7 was an independent biomarker in patients with ACS after PCI.

**Conclusions:**

SNHG7 might be a diagnostic and prognostic tool for ACS patients.

## Background

Coronary heart disease (CHD) is a pivotal reason for reduced quality of life, disability, and death [1]. The most dangerous and deadly form of CHD is acute coronary syndrome (ACS) [2]. ACS is a series of clinical syndromes caused by rupture or erosion of atherosclerotic plaques, resulting in narrowing or occlusion of the coronary artery lumen, causing acute ischemia or necrosis of the myocardium [3]. ACS has a poor prognosis and a high mortality rate among cardiology diseases. Currently, most patients with ACS are treated with percutaneous coronary intervention (PCI) [4]. The main application of this method is cardiac catheterization technology, which can make the narrowed or occluded coronary artery lumen reopen and improve the quality of the coronary artery [5]. Early PCI treatment can improve ischemia, save dying myocardium, and improve life quality and clinical prognosis [6]. However, there are corresponding risks during the procedure, such as exacerbated rupture of coronary plaques, abnormal endothelial proliferation, restenosis, and acute thrombosis. Due to the rapidly changed clinical condition and poor predictability, a high mortality rate exists in the postoperative period. Based on this, it is important to seek factors influencing prognosis after PCI.

LncRNAs are widely involved in cardiovascular diseases and pathological conditions, and their expression levels change, making them valuable as biomarkers [7, 8]. The expression of lncRNA FENDRR is declined in patients with unstable angina (UA) and ST-segment elevation myocardial infarction (STEMI), supporting that this lncRNA may serve as a non-invasive marker [9]. The clinical value of BACE1-AS is reflected by its prognostic significance in forecasting major adverse cardiovascular events (MACEs) for patients with ACS [10]. Importantly, in the mice with myocardial infarction, the expression of small nuclear RNA host gene 7 (SNHG7) is facilitated and its depletion exerts a beneficial function in myocardial infarction [11]. It is known that acute myocardial infarction is a subtype of ACS, thus, SNHG7 may be involved in ACS.

Considering the discovery before, it was supposed that SNHG7 might be abnormally expressed in ACS, which may contribute to the predictive and diagnostic identification of ACS. The ACS patients were volunteers for the research of SNHG7. Specifically, the expression of SNHG7 was assessed and its diagnostic importance was unveiled. The correlation between SNHG7 and ACS was revealed. The prognostic value of SNHG7 on the MACE and survival probability was underlined.

## Methods

### Included patients

A retrospective study was designed under the approval of the Ethics Committee of JiuJiang NO.1 People’s Hospital (ethical code: 2018-031). 169 patients with ACS who were admitted to JiuJiang NO.1 People’s Hospital in November 2018 to October 2021 and underwent PCI for the first time were selected as study subjects, no patients received any therapy prior to sample collection. Inclusion criteria: (1) meet the diagnosis of UA, non-ST-segment elevation myocardial infarction (NSTEMI) and STEMI [12, 13]; (2) the postoperative blood flow of the target vessel reaching thrombolysis in myocardial infarction (TIMI) grade 3; (3) complete clinical history and (4) no contraindication related to surgery. Exclusion criteria: (1) patients with congenital heart disease, heart failure, and peripheral vascular diseases; (2) history of previous cardiac surgery, like coronary stent implantation and coronary artery bypass; (3) patients with a recent history of infectious diseases; (4) patients with missing case data, and those who did not cooperate with the follow-up visit. 77 healthy controls were selected at the same time as the physical examination population, with similar age and sex to the experimental group, no symptoms of chest tightness and chest pain, and no obvious abnormalities in biochemical tests, electrocardiogram, chest CT, cardiac color Doppler ultrasound and other imaging tests. The patients and healthy controls signed informed consent.

### Serum collection

The venous blood was gathered immediately from patients on the day of admission. The blood from control individuals was gathered on the morning after fasting. The blood samples were stood at room temperature. The upper solution was collected and deposited in a -80℃ refrigerator.

### Clinicopathological information and follow-up

All patients received coronary angiography and the Gensini scores were estimated to reflect the severity of lesions in the coronary artery. General information such as age, BMI, and gender and clinical data such as auxiliary examination was collected by asking patients and retrieving medical records.

The duration of follow-up in this study was one year, and follow-up was conducted in the clinic, by telephone, and in subsequent visits. The death endpoint was defined as all-cause mortality. The MACE of all patients included all-cause death, heart failure, angina recurrence, non-fatal myocardial infarction, major arrhythmia, and repeat revascularization [14].

### Assessment of SNHG7 expression

The serum/plasma RNA isolation kit (HYCEZMBIO, Wuhan, China) was used for collecting RNA specimens. The concentration and purity of isolated RNA by the method of measuring absorbance at 260 nm and 280 nm by using the Agilent 2100 Bioanalyzer (Agilent, Santa Clara, CA, USA), and the 260/280 nm ratio of total RNA used in the study was 1.8-2.0. The optimized buffer system enables efficient and specific binding of RNA from the sample to the silica matrix centrifugal adsorption column. High-purity RNA was obtained by a two-step wash followed by elution using ddH_2_O. The RevertAid RT kit (Thermo Scientific, Waltham, America) was combined with total RNA to synthesize cDNA. The GAPDH was a reference. The primers of genes were purchased from Sangon Biotech (Shanghai, China). The primer sequences of lncRNA SNHG7 were shown as follows: lncRNA-SNHG7 primers forward: 5’-GTCAGCCGCATCTTCTTTTG-3’, reverse: 5’-GCGCCCAATACGACCAAATC-3’; GAPDH primers forward: 5’-CGCTCTCTGCTCCTCCTGTTC-3’, reverse: 5’-ATCCGTTGACTCCGACCTTCAC-3’. The biomarker 2X SYBR Green Fast qPCR mix (Beijing, China) was used for expression detection. The machine used was ABI 7700. PCR cycling included an initial denaturation for 2 min at 95 ˚C, followed by 40 cycles of 94 ˚C for 20 s, 60 ˚C for 40 s. Three technical replicates were conducted. The quantification of SNHG7 was reckoned by the 2^−∆∆CT^ formulas.

### Statistical analysis

All statistical analyses and plotted tables were done in GraphPad Prism 7 and SPSS 20.0 software. For data analysis, the measurement data were expressed as mean ± standard deviation and the count data were expressed as constitutive ratio (%). T-test was used for difference comparison of measurement data between two groups, while one-way ANOVA followed by Tukey’s test was used to compare the difference among multiple groups (> 2). The chi-square test was used to compare the count data between groups. Pearson’s method was used to analyze various correlations. *p* < 0.05 indicated that the differences were statistically different. To evaluate the predictive value of SNHG7 expression changes in ACS, the ROC was used. To evaluate the predictive effect of SNHG7 on MACE and survival, Kaplan-Meier (K-M) curves and Cox regression analyses were used. In the cox regression analysis, MACE and survival were aet as dependent variables, while age, BMI, gender, hypertension, diabetes, history of family, TC, TG, LDL, HDL, CK-MB, cTnT, Gensini score and SNHG7 were used as the dependent variable.

## Results

### Clinicopathological features of ACS patients

A total of 245 subjects were enrolled, including 169 ACS patients and 77 controls. The ACS group was further subdivided into 65 NSTEMI patients, 50 SETMI patients, and 54 UA patients. Their basic clinical data and biochemical characteristics were summarized in Table 1. There were no significant differences in age, BMI, gender, hypertension, diabetes, and history of family between the ACS group and the control group (*P* > 0.05). The ACS group had a significantly higher TC, TG, LDL, CK-MB, and cTnT than the control group (*P* < 0.001). The HDL levels were lower in the ACS group than in the control group (*P* < 0.05). The average Gensini score of ACS patients was 48.30 ± 14.96.

**Table 1:** Clinicopathological factors of healthy controls and ACS patients

Factors	Healthy controls (*N* = 77)	ACS patients (*N* = 169)	*P* value
Age, year	51.46 ± 5.62	52.07 ± 6.37	0.473
BMI, kg/m^2^	24.87 ± 3.07	25.12 ± 3.28	0.570
Gender, Male (%)	39 (51.3)	92 (54.4)	0.650
Hypertension, N (%)	19 (25.0)	56 (33.1)	0.201
Diabetes, N (%)	18 (23.7)	44 (26.0)	0.695
History of family, N (%)	9 (11.8)	36 (21.3)	0.077
TC, mmol/L	4.30 ± 0.87	4.79 ± 0.91	< 0.001
TG, mmol/L	1.37 ± 0.60	1.80 ± 0.65	< 0.001
LDL, mmol/L	2.78 ± 0.90	3.29 ± 0.88	< 0.001
HDL, mmol/L	1.18 ± 0.25	1.07 ± 0.42	0.032
CK-MB, U/ml	15.86 ± 4.38	49.71 ± 14.49	< 0.001
cTnT, ng/L	22.16 ± 7.02	158.37 ± 29.03	< 0.001
Gensini score	/	48.30 ± 14.96	< 0.001

*Abbreviations* BMI, body-mass index; TC, total cholesterol; TG, triglyceride; LDL, low density lipoprotein; HDL, high density lipoprotein cholesterol; CM-MB, creatine kinase- myocardial band; cTnT, cardiac troponin T

### The expression of SNHG7 in ACS

**Fig. 1:**
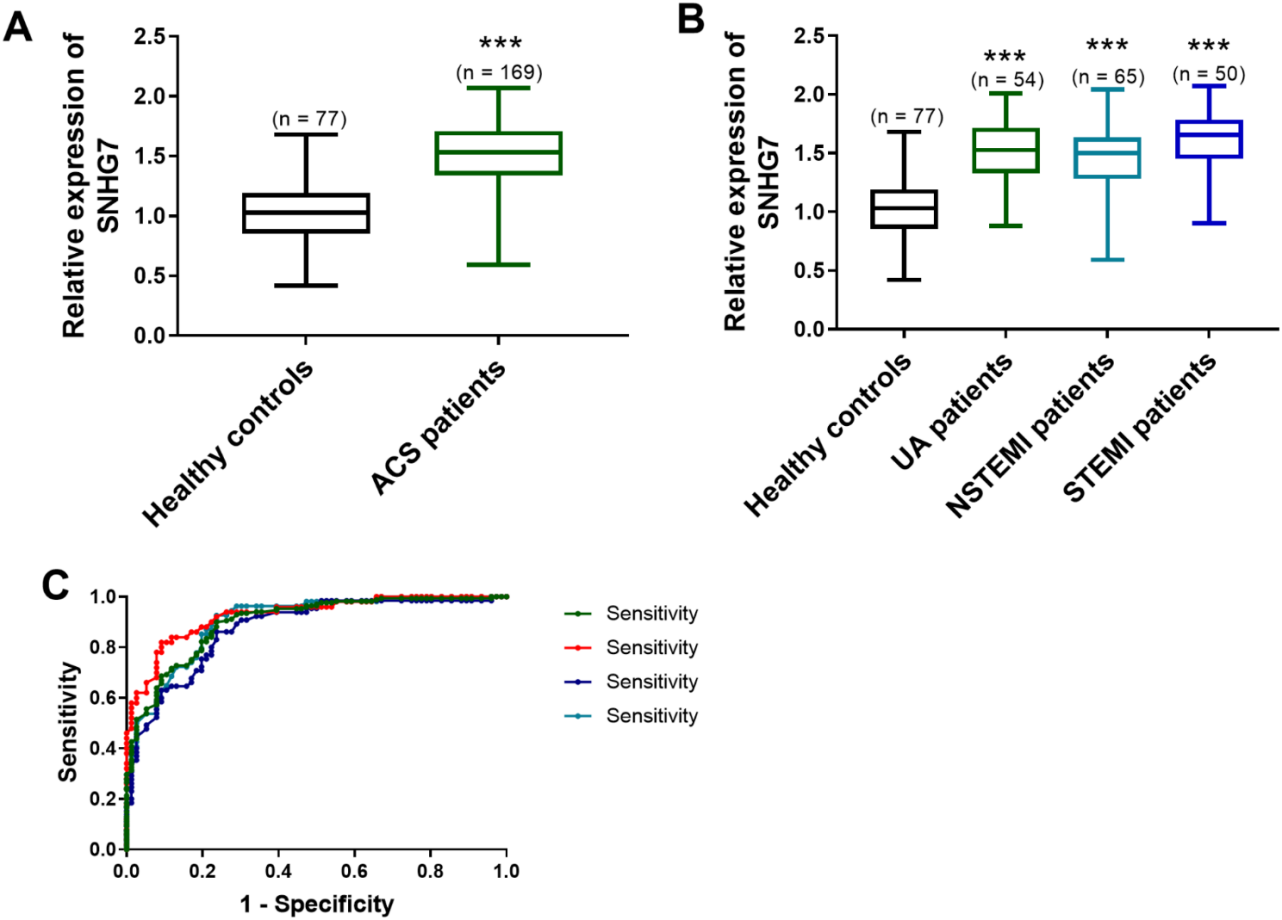
Upregulation of SNHG7 and its clinical significance. **(A)** The increase of SNHG7 in ACS. **(B)** The expression of SNHG7 in the subtypes of ACS. **(C)** The ROC curves of SNHG7 in diagnosis. ***P < 0.001, compared to healthy controls

The quantification of SNHG7 in the ACS patients was elevated compared to healthy controls, supporting that SNHG7 was implicated in the pathological progression of ACS (*P* < 0.001, Fig. 1A). The concentration of SNHG7 was increased in UA patients, NSTEMI patients, and STEMI patients (*P* < 0.001, Fig. 1B). No discrepancy in SNHG7 expression was observed among UA patients, NSTEMI patients, and STEMI patients (*P* > 0.05, Fig. 1B).

### The predictive significance of SNHG7

The clinical prediction of SNHG7 on ACS and its subtypes was estimated by plotting ROC curves. The AUC was 0.900, 0.904, 0.874, 0.927 on predicting ACS, UA, NSTEMI, and STEMI (Fig. 1C), suggesting that SNHG7 had values in predicting ACS and subtypes of ACS from healthy individuals.

### SNHG7 associated with ACS

**Fig. 2:**
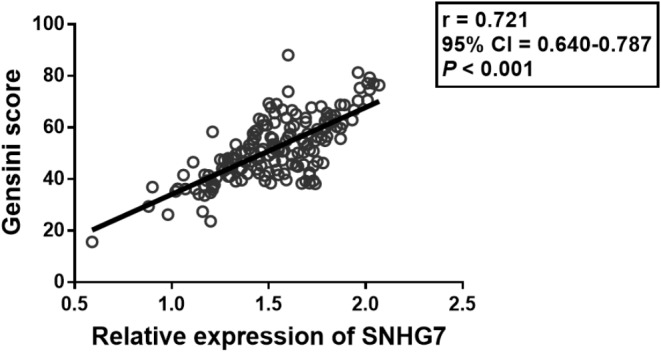
The strong positive association between Gensini score and SNHG7

Gensini is a common marker in estimating the severity of coronary lesions [15]. The expression of SNHG7 was strongly positively correlated with the Gensini score (Fig. 2), reflecting that SNHG7 might associate with the severity of ACS.

### SNHG7 forecasted MACE probability

**Fig. 3:**
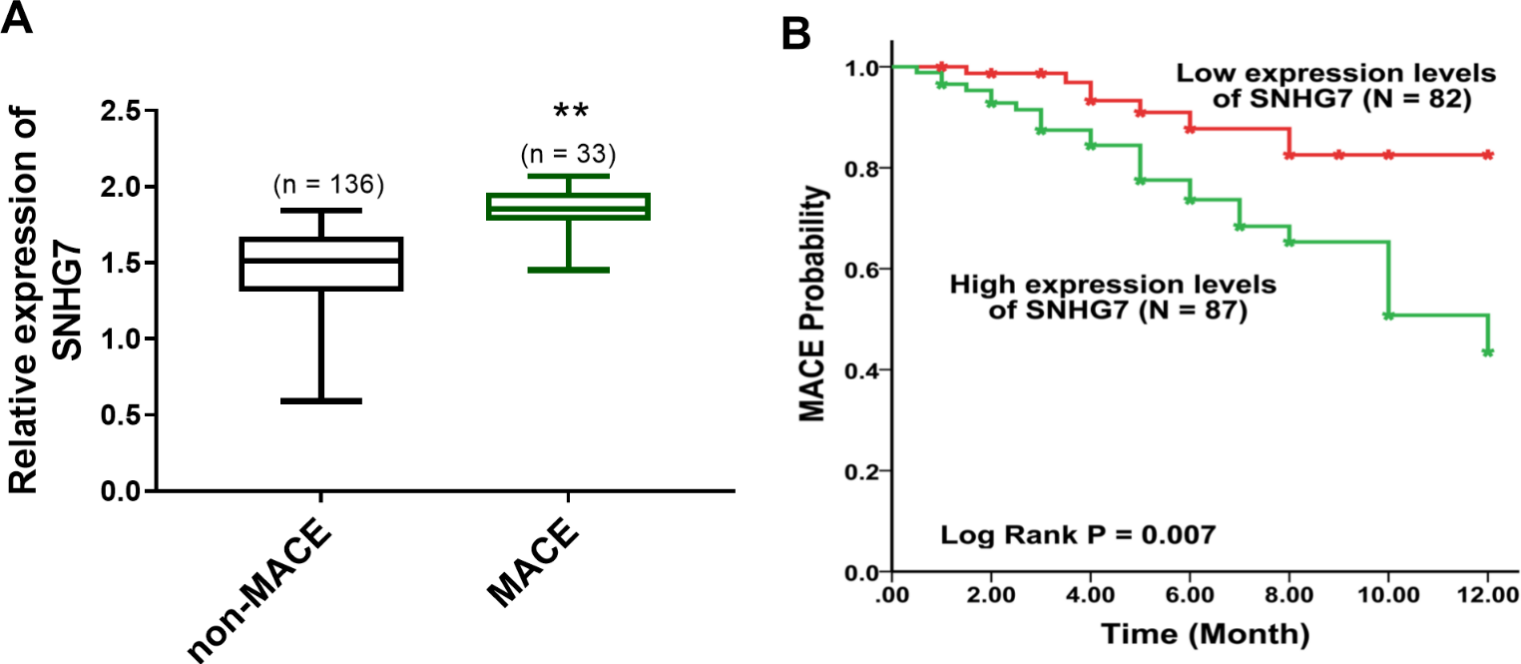
The function of SNHG7 on MACE probability. **(A)** SNHG7 was upregulated in the MACE group. **(B)** The K-M curve of SNHG7 for MACE probability. **P < 0.01, compared to the non-MACE group

All patients with ACS were catalogued into the non-MACE group and MACE group according to the final situation of patients. At the one-year follow-up event, 136 patients had no MACE events and 33 patients had MACE events. The expression of SNHG7 was increased in the MACE group compared with the non-MACE group (*P* < 0.01, Fig. 3A), indicating that the expression of SNHG7 might associate with the development of ACS.

Based on the median expression of SNHG7, 82 patients were catalogued in the low-expression group and 87 patients were catalogued into the high-expression group. The K-M curve documented that patients with high expression levels of SNHG7 were more likely to have MACE compared to those with low expression (*P* = 0.007, Fig. 3B). The Cox regression method further analyzed the independent risks of poor diagnostic outcomes. Gensini score (HR = 2.564, 95% CI = 1.178–5.579, *P* = 0.018, Table 2) and relative expression of SNHG7 (HR = 3.037, 95% CI = 1.254–7.357, *P* = 0.014, Table 2) were independent indicators for predicting MACE.

**Table 2:** Cox regression analysis of risk factors for MACE

Factors	HR	95% CI	*P* value
Age, year	1.270	0.595–2.709	0.536
BMI, kg/m^2^	0.764	0.356–1.64	0.490
Gender, Male (%)	1.108	0.518–2.373	0.791
Hypertension, N (%)	0.728	0.321–1.653	0.448
Diabetes, N (%)	1.130	0.417–3.056	0.810
History of family, N (%)	0.721	0.314–1.658	0.442
TC, mmol/L	0.756	0.356–1.601	0.464
TG, mmol/L	1.580	0.732–3.409	0.244
LDL, mmol/L	1.465	0.647–3.317	0.360
HDL, mmol/L	0.692	0.321–1.493	0.348
CK-MB, U/ml	0.716	0.340–1.508	0.379
cTnT, ng/L	0.609	0.294–1.259	0.181
Gensini score	2.564	1.178–5.579	0.018
Relative expression of SNHG7	3.037	1.254–7.357	0.014

*Abbreviations* MACE, major adverse cardiovascular events; HR, hazard ratio; CI, confidence interval; BMI, body-mass index; TC, total cholesterol; TG, triglyceride; LDL, low density lipoprotein; HDL, high density lipoprotein cholesterol; CM-MB, creatine kinase- myocardial band; cTnT, cardiac troponin T

### SNHG7 forecasted survival probability

**Fig. 4:**
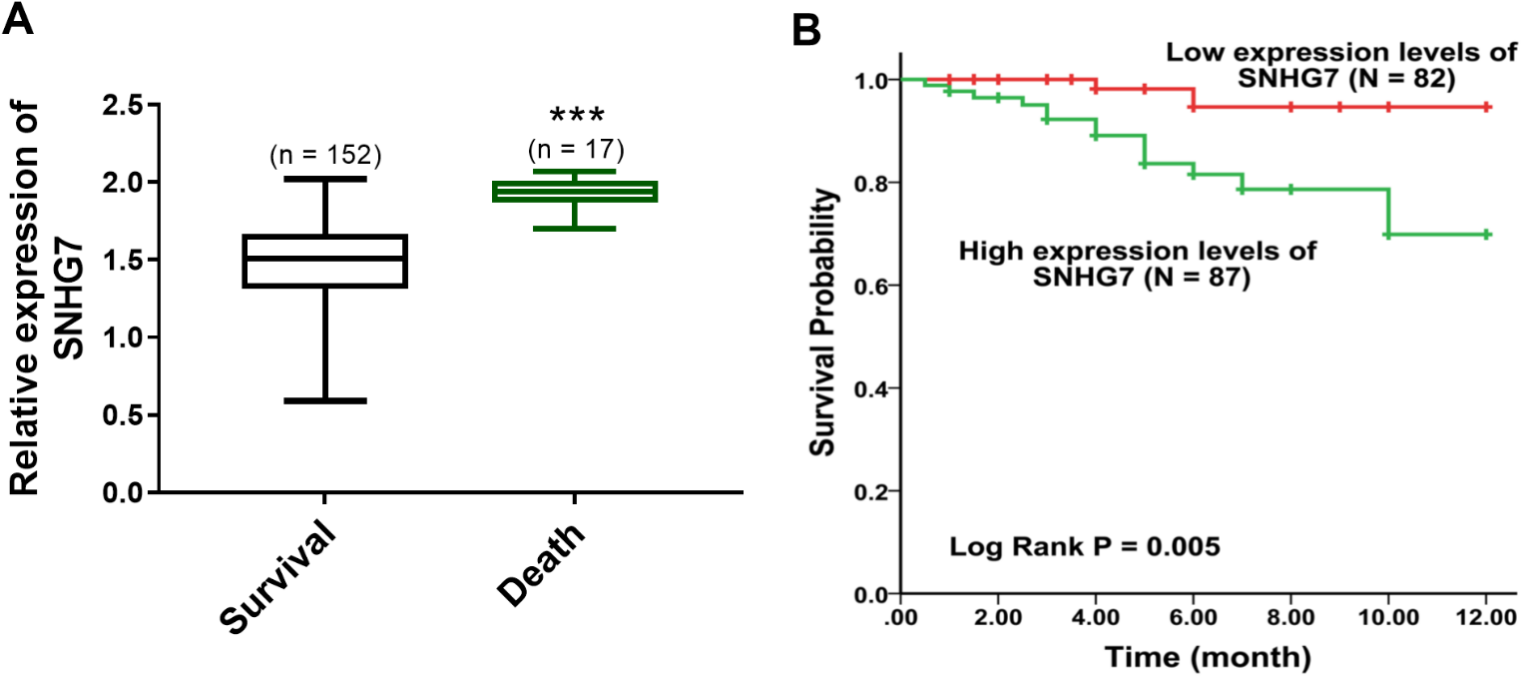
The prognostic role of SNHG7. **(A)** A high level of SNHG7 in the death group. **(B)** The K-M curve of SNHG7 on predicting the survival probability. ***P < 0.001, compared to the survival group

The patients were categorized into a death group (*N* = 17) and a survival group (*N* = 152) according to their death status within one year. SNHG7 was upregulated in the death group (*P* < 0.001, Fig. 4A), which pinpointed that the upregulation of SNHG7 might be involved in the development of ACS.

The K-M curve was plotted to emphasize the survival distribution of patients with different expression levels. The patients with high expression of SNHG7 had lower survival probability (*P* = 0.005, Fig. 4B), supporting that SNHG7 might be a prognostic predictor. The Cox results further emphasized that the expression of SNHG7 (HR = 3.037, 95% CI = 1.555–34.036, *P* = 0.012, Table 3) was an independent biomarker.

**Table 3:** Cox regression analysis of risk factors for survival

Factors	HR	95% CI	*P* value
Age, year	1.713	0.567–5.175	0.340
BMI, kg/m^2^	0.764	0.259–2.283	0.635
Gender, Male (%)	1.108	0.665–6.277	0.212
Hypertension, N (%)	0.728	0.214–2.314	0.563
Diabetes, N (%)	1.130	0.222–3.911	0.923
History of family, N (%)	0.721	0.224–2.214	0.549
TC, mmol/L	0.756	0.342–3.017	0.977
TG, mmol/L	1.580	0.413–3.443	0.746
LDL, mmol/L	1.465	0.549–6.322	0.318
HDL, mmol/L	0.692	0.141–1.444	0.180
CK-MB, U/ml	0.716	0.410–3.861	0.688
cTnT, ng/L	0.609	0.145–1.414	0.173
Gensini score	2.564	0.772–6.885	0.135
Relative expression of SNHG7	3.037	1.555–34.036	0.012

*Abbreviations* HR, hazard ratio; CI, confidence interval; BMI, body-mass index; TC, total cholesterol; TG, triglyceride; LDL, low density lipoprotein; HDL, high density lipoprotein cholesterol; CM-MB, creatine kinase- myocardial band; cTnT, cardiac troponin T

## Discussion

ACS has become the most common cardiovascular emergency and critical illness [16]. Accurate diagnosis and early intervention are essential to improve the prognosis of patients and reduce the mortality rate of patients. PCI is fundamental management to reopen the narrowed or occluded coronary artery lumen and to improve myocardial perfusion [17]. PCI can only change the morphology of the coronary artery lumen, but not the risk factors that cause the disease. Because of the persistence of underlying etiologic factors and external risk factors, there is a high risk of recurrent vascular occlusion and other prognostic problems after the procedure. Therefore, a continued search for markers associated with the development of ACS is warranted.

LncRNAs are widely involved in cardiovascular disease, and their expression changes in different pathological conditions, thus having value as non-invasive diagnostic markers [18]. The increase of lncRNA NORAD reflects aggravated stenosis degree, a severe inflammatory disorder in patients with coronary heart disease, predicting the predictive possibility of NORAD [19]. In coronary artery disease, elevated levels of lncRNA Coromarker are linked with disease severity [20]. As mentioned above, the alternation of lncRNAs is associated with the progression of cardiovascular diseases. In the present paper, the expression of SNHG7 and its clinical significance in ACS were assessed. The expression of SNHG7 was upregulated in all ACS patients, indicating that ACS appeared to influence the expression of this lncRNA. The expression of SNHG7 was increased in UA patients, NSTEMI patients, and STEMI patients, which was in line with the results of ACS. Zhang et al. illustrate the upregulation of SNHG7 in atherosclerosis and the mechanism of SNHG7 in biological functions in atherosclerosis [21]. The upregulation of SNHG7 in atherosclerosis was consistent with our findings. The predictive possibility of SNHG7 was estimated by plotting ROC curves. The result indicated that SNHG7 exerted function in predicting ACS, UA, NSTEMI, and STEMI from healthy individuals.

The high Gensini score is an indicator of the severity and complexity of ACS [22]. In this research, the high expression of SNHG7 was closely associated with the increase in Gensini scores, indicating that SNHG7 might have a relationship with the severity of ACS. The abnormally expression lncRNAs is observed as a predictor for the prognosis of cardiovascular illnesses [23, 24]. For instance, lncRNA-Ang362 is correlated with the poor overall outcome of patients with coronary heart disease after PCI [25]. High expression of lncRNA PELATON reveals the high incidence of MACE and poor diagnosis of ACS [26]. The change of SNHG7 is verified as a predictive tool in several disorders, such as neonatal sepsis, hepatocellular carcinoma, and colon adenocarcinoma [27–29]. In this current exploration, the expression of SNHG7 was elevated in MACE compared to non-MACE patients and the death group compared to the survival group, suggesting that SNHG7 was overexpressed in ACS patients with poor diagnosis. The findings of the K-M curve revealed that the patients with high expression levels of SNHG7 appeared to suffer a high rate of poor prognosis, supporting that SNHG7 might serve as a prognostic predictor. The Cox regression method found that SNHG7 was an independent risk predictor of ACS, indicating the prognostic possibility of SNHG7. It is known that, SNHG7 plays an important role in cardiovascular disease [30, 31]. In hypoxia/reoxygenation (H/R)-induced cardiomyocyte injury, elevated SNHG7 is related to the activation of cardiomyocyte oxidative stress and apoptosis, and miR-181b-5p is identified to be its downstream target [32]. Besides, in mice myocardial infarction models, overexpressed SNHG7 is also detected, which contributes to cardiac fibroblasts apoptosis, fibrosis and inflammation [11]. The regulatory role of SNHG7 in cardiomyocyte and cardiac fibroblasts functions might be its underlying mechanism in ACS. Limitations exist in this dissertation, such as a small sample size, relatively short-term follow-up, and a retrospective study design. Further large-sample, prospective experiments are needed to improve the clinical significance of this trial. In addition, the regulator mechanism of SNHG7 in ACS should be explored in vivo or in vitro.

## Conclusions

In summary, the expression of SNHG7 was upregulated in ACS patients, UA patients, NSTEMI patients, and STEMI patients. The overexpression of SNHG7 could predict these kinds of diseases in healthy subjects. A close association between SNHG7 and Gensini score was observed in ACS patients. The upregulation of SNHG7 was found in the MACE group, which could function as a diagnostic tool for MACE occurrence. SNHG7 was highly expressed in the death group and its increase indicated a high mortality for ACS patients. This paper might provide a reference for guiding clinical decision-making for PCI and the treatment of ACS.

## Data Availability

The datasets used and/or analysed during the current study are available from the corresponding author on reasonable request.

## Abbreviations

ACS: Acute coronary syndrome
CHD: Coronary heart disease
PCI: Percutaneous coronary intervention
UA: Unstable angina
STEMI: ST-segment elevation myocardial infarction
MACEs: Major adverse cardiovascular events
SNHG7: Small nuclear RNA host gene 7
NSTEMI: Non-ST-segment elevation myocardial infarction
TIMI: Thrombolysis in myocardial infarction
